# Molecular Image-Guided Theranostic and Personalized Medicine 2014

**DOI:** 10.1155/2015/258612

**Published:** 2015-03-25

**Authors:** Hong Zhang, Mei Tian, Ignasi Carrio, Ali Cahid Civelek, Yasuhisa Fujibayashi

**Affiliations:** ^1^The Second Affiliated Hospital of Zhejiang University, Hangzhou, Zhejiang, China; ^2^Hangzhou Binjiang Hospital of Zhejiang University, Hangzhou, Zhejiang, China; ^3^San Paul Hospital, Autonomous University of Barcelona, Barcelona, Spain; ^4^NIH Clinical Center, Bethesda, MD, USA; ^5^National Institute of Radiological Sciences, Chiba, Japan

Molecular image-guided therapy has been demonstrated to be effective in improving diagnosis, prognosis, planning, and monitoring of personalized medication. Molecular imaging modalities include positron emission tomography (PET), single-photon emission-computed tomography (SPECT), magnetic resonance imaging (MRI), computed tomography (CT), ultrasound (US), and optical imaging (Ramen, quantum dots, and bioluminescence). Among clinical molecular imaging modalities, radionuclide imaging technique is the most sensitive one which could provide target-specific information as well as function, pathway activities, and cell migration in the intact organism. For instance, the radiotracer could noninvasively assess diseases treatment endpoints which are used to rely almost exclusively on biopsies and histopathological assays. New leads on the development of personalized theranostic (image and treat) agents would allow for more accuracy in the selection of patients who may respond to treatment.

Topics covered in this special issue include advances in molecular imaging modalities in oncological disease management, image-guided approach of brain function, stem cell technology, and receptor based imaging approach. For example, Q. Wang's group at Nanfang Hospital of Southern Medical University reported a retrospective study on 51 consecutive patients to investigate what causes the false negative of adenocarcinoma with BAC features on ^18^F-FDG PET/CT. Their study demonstrated that different types of adenocarcinoma with BAC features exhibited different ^18^F-FDG uptake patterns. False negative on ^18^F-FDG PET/CT mainly occurs in those lesions presented as the nonsolid nodule on CT, but not the solid nodule. Y. Dong and her coauthors from Zhejiang University evaluated the efficacy of PET/CT in association with serum tumor maker assays in the follow-up of patients with breast cancer (BC). This study indicated that PET/CT was a highly efficient tool to follow-up patients with BC compared with CITs in terms of sensitivity and specificity, allowing for the detection of metastatic and/or recurrent cases. The high serum levels of CA 15-3 in confirmed positive PET/CT patients compared to negative ones indicated the clinical value of CA 15-3 in BC follow-up. H. Hou and his coauthors from the same institute reported a pilot study on the prognostic value of ^99m^Tc-pertechnetate thyroid scintigraphy for predicting the outcomes of fixed dose (5 mCi) of radioiodine. A fixed dose of 5mCi radioiodine seems to be practical and effective for treating GD patients with thyroid mass ≤40.1 g and ^99m^Tc-pertechnetate uptake ≤15.2%. This study demonstrates that ^99m^Tc-pertechnetate thyroid scintigraphy is an important prognostic factor for predicting the outcomes of RIT.

Mesenchymal stem cells (MSCs) have been proposed as a promising cell population for cell therapy and regenerative medicine applications. However, the low retention and poor survival of engrafted cells hampered the therapeutic efficacy of engrafted MSCs. In this issue, F. Cao and her coauthors from Fourth Military Medical University and Chinese PLA General Hospital presented their work on the protective effects of ghrelin on engrafted adipose derived mesenchymal stem cells (ADMSCs) and its beneficial effects with cellular therapy in mice myocardial infarction (MI). Their study revealed that ghrelin may serve as a promising candidate for hormone-driven approaches to improve the efficacy of mesenchymal stem cell-based therapy for cardiac ischemic disease via PI3K/AKT pathway.

Recently, a number of tracers have been introduced due to the encouraging results from the applications of radiolabeled ligand-receptor system. The current status of somatostatin receptor based imaging and radionuclide therapy is summarized and discussed in the review paper by C. Xu and her coauthor. The* hSSTr2* gene can act as not only a reporter gene for in vivo imaging, but also as a therapeutic gene for local radionuclide therapy. Even a second therapeutic gene can be transfected into the same tumor cells together with* hSSTr2* reporter gene to obtain a synergistic effect.

Neuroscience is a hot topic in recent years. Maladaptive use of the Internet results in Internet addiction (IA), which is associated with various negative consequences. Molecular and functional imaging approaches have been increasingly used for the analysis of neurobiological and neurochemical changes of the brain. Y. Zhu and his coauthors summarize the molecular and functional imaging findings on neurobiological mechanisms of IA, focusing on magnetic resonance imaging (MRI) and nuclear imaging modalities including positron emission tomography (PET) and single-photon emission-computed tomography (SPECT). MRI studies demonstrate that structural changes in frontal cortex are associated with functional abnormalities in Internet addicted subjects. Nuclear imaging findings indicate that IA is associated with dysfunction of the brain dopaminergic systems. Abnormal dopamine regulation of the prefrontal cortex (PFC) could underlie the enhanced motivational value and uncontrolled behavior over Internet overuse in addicted subjects.

In summary, molecular imaging could be applied to target characterization, underlying disease progression and evaluation of therapeutic response and stem cell and brain function. This special issue provides a platform of the efficacy of personalized medication from molecular imaging technology which may have high impact on patient care.

*Hong Zhang* *Mei Tian* *Ignasi Carrio* *Ali Cahid Civelek* *Yasuhisa Fujibayashi*

